# Delivery of siRNA Using Functionalized Gold Nanorods Enhances Anti-Osteosarcoma Efficacy

**DOI:** 10.3389/fphar.2021.799588

**Published:** 2021-12-20

**Authors:** Man Zhang, Jinti Lin, Jiakang Jin, Wei Yu, Yiying Qi, Huimin Tao

**Affiliations:** ^1^ Department of Orthopedic Surgery, the Second Affiliated Hospital, Zhejiang University School of Medicine, Hangzhou, China; ^2^ Orthopedics Research Institute of Zhejiang University, Hangzhou, China; ^3^ Key Laboratory of Motor System Disease Research and Precision Therapy of Zhejiang Province, Hangzhou, China

**Keywords:** gold nanorods, photothermal therapy, RNA interference, osteosarcoma, autophagy

## Abstract

Gold nanorods (GNRs) are intensively explored for the application in cancer therapy, which has motivated the development of photothermal therapy (PTT) multifunctional nanoplatforms based on GNRs to cure osteosarcoma (OS). However, the major limitations include the toxicity of surface protectants of GNRs, unsatisfactory targeting therapy, and the resistant effects of photothermal-induced autophagy, so the risk of relapse and metastasis of OS increase. In the present study, the GNR multifunctional nanoplatforms were designed and synthesized to deliver transcription factor EB (TFEB)-siRNA–targeting autophagy; then, the resistance of autophagy to PTT and the pH-sensitive cell-penetrating membrane peptide (CPP) was weakened, which could improve the tumor-targeting ability of the GNR nanoplatforms and realize an efficient synergistic effect for tumor treatment. Meanwhile, it is worth noting that the GNR nanoplatform groups have anti-lung metastasis of OS. This study provides a new reference to improve the efficacy of OS clinically.

## Introduction

Surgical resection of solid tumors and postoperative chemotherapy and radiotherapy are currently adopted for the treatment of malignant tumor, but the side effects of chemotherapeutic drugs and the development of resistant tumor cells lead to patient intolerance, tumor recurrence, and distant metastases (Holohan et al., 2013; Wyld et al., 2015; Zugazagoitia et al., 2016). Photothermal therapy (PTT) is a promising and effective approach in cancer therapy, and it applies light-absorbing nanomaterials to convert light radiation into heat so as to kill tumor cells (Riley and Day 2017). The approach displays many advantages, including high penetration depth in the biological tissues, minimal invasiveness to normal tissues, high spatial precision, and so on (Xu et al., 2020). It has been studied that NIR triggers nanomaterial-induced PTT that could be used as an effective way to treat deep tissue pancreatic cancer and gallbladder disease (Ma et al., 2018; Cai et al., 2020; Jia et al., 2020). PTT also could enhance the antitumor immune response after the highly immunogenic thermal death of tumor cells (Liu et al., 2018) and promote tumor infiltration of CAR T cells (Chen et al., 2019), which makes. These characteristics of PTT enable it suitable for the treatment of some highly malignant and aggressive metastatic tumors that are difficult to be completely removed with surgery, such as osteosarcoma (OS). For the malignant tumor with the highest incidence in the skeletal system (Luetke et al., 2014), a major problem, as yet unsolved, is that patients with relapsed or unresectable OS have dismal prognosis (Ritter and Bielack 2010; Harrison et al., 2018). A recent study has revealed that PTT inhibited the tumor growth in an OS mouse model (Xiong et al., 2021). However, the nanomaterial-triggered PTT could not exhaustively eradicate the tumor for the nonuniform distribution of hyperthermia (Zhang et al., 2013; Li et al., 2015) and the production of resistant tumor cells. Therefore, the development of an advanced PTT-based strategy with a stable photothermal conversion rate could improve cancer cell killing *via* countering resistance, with great potential for clinical transformation.

**Graphical Abstract:**
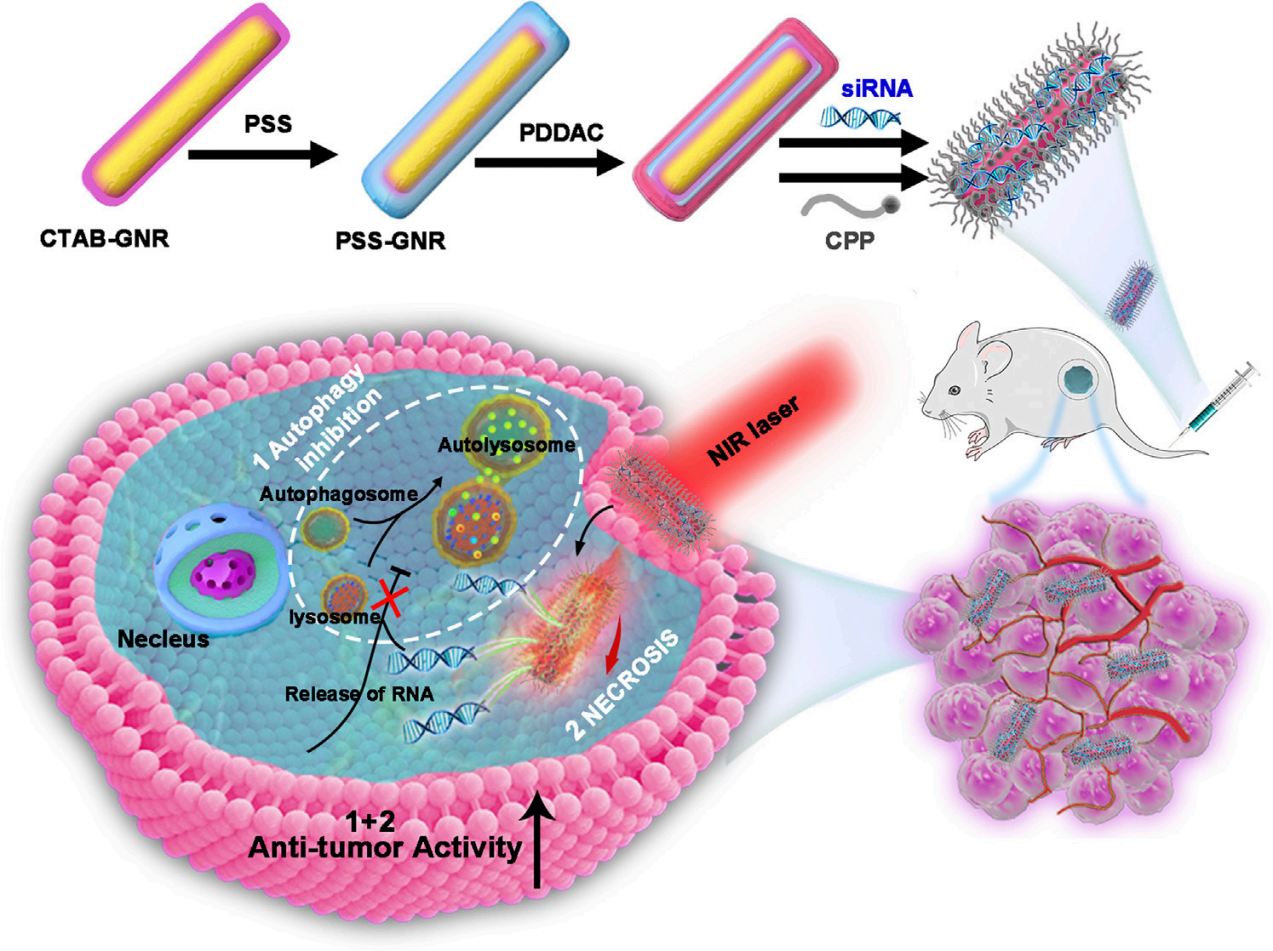
Schematic representation of the photothermal therapy approach using GNR@siRNA/CPP multifunctional nanoplatforms in osteosarcoma.

In the process of photothermal treatment of the tumor, the photothermal absorption capacity is highly dependent on the photothermal conversion rate of the photothermal material and the laser wavelength (Liu et al., 2017). With prominent optical absorption and excellent photothermal conversion efficiency in the near-infrared (NIR) window, MoS_2_-based nanocomposites can be used for cancer therapy. However, it is still difficult to synthesize stable MoS_2_ nanocomposites that may also interfere with the reproductive system (Ai et al., 2021). Another nanomaterial, namely, copper selenide, has broad application prospects in the field of near-infrared thermal ablation for its localized surface plasmon resonance (LSPR) in the near-infrared region. But copper selenide is easy to be oxidized to release copper ions, thus causing LSPR changes, and trigger adverse effects *in vivo*, and these characteristics limit its clinical application (Wang et al., 2021). Gold nanorods (GNRs) have a mature synthetic method without genotoxicity (Zarska et al., 2018). Meanwhile, GNRs have been explored as light-absorbing nanomaterials for cancer therapy for their unique biological characteristics, strong absorption of light in the near-infrared region, and excellent photothermal conversion (Huang et al., 2009). The size of the gap between tumor vascular endothelial cells and normal vascular endothelial cells is different, and thus, the number of nanocarriers was designed to enhance passive accumulation and extend blood circulation at tumor sites by increasing permeability and retention (EPR) (Golombek et al., 2018) effects, which could weaken cellular uptake, in turn.

A cell-penetrating membrane peptide (CPP) is a small molecule polypeptide which can carry various cargoes across the cellular membranes in an intact, such as liposomes, full-length proteins, nucleic acids, and nanoparticles (Taylor and Zahid 2020). Studies have confirmed that CPP-functionalized gold nanorods could improve the cell penetration, exert excellent efficacy (Riveros et al., 2020), and improve the targeting of tumor cells (Tan et al., 2017). The characteristic features of acidosis in tumor microenvironments are taken as advantages to synthesize pH-responsive CPP and realize the targeting of therapeutic drugs to tumor tissue, and it is undoubtedly a strategy for the treatment of tumors. One study pointed out that pH-controllable CPP provided an active cell-penetrating characteristic in tumors (Lee et al., 2017), while the role of CPP-functionalized gold nanorods in the treatment of OS was rarely been reported.

As a physiological activity of cells to maintain substance metabolism, internal environment stability, and genome integrity, autophagy also plays an important role in the abnormal proliferation of tumor cells (Levy et al., 2017). The activation of autophagy helps to remove damaged and aging organelles timely and maintain the continuous abnormal proliferation of tumor cells (Ravanan et al., 2017). In addition, the development of drug resistance in cancer cells has become a major barrier for the effective treatment of cancer with chemotherapy, and autophagy has been proved to be involved in the production of resistant cancer cells (Li et al., 2017; Taylor et al., 2018). In addition, the activation of autophagy provides necessary energy and nutritional supply for DNA repair and delays cell apoptosis following photothermal therapy (Wu et al., 2018; Zhang et al., 2019). On the other hand, the surface chemistry of GNRs mediates their biological toxicity, and CTAB-GNRs induce cell apoptosis and autophagy by damaging the mitochondria and activating intracellular reactive oxygen species (ROS) (Wan et al., 2015). Research studies have confirmed that near-infrared photothermal therapy using gold nanoparticles could increase autophagic cell death in breast cancer (Zhang et al., 2017), and the autophagy suppressor enhances the efficacy of PTT in tumor cells (Ma et al., 2018). In the process of clinical application, near-infrared photothermal therapy could overcome autophagy-induced resistance to PTT and chemotherapy in the treatment of tumors, increase the dose of chemotherapeutic drugs, the treatment temperature, or extend the irradiation time, which may result in unpredictable damage to normal tissues. Gene silencing is a promising technique for cancer treatment with precision and few side effects. The silencing of the transcription factor EB (TFEB) gene has positive effects on autophagy inhibition and antitumor activities (Golombek et al., 2018; Li et al., 2019; Astanina et al., 2021). However, the renal clearance, nuclease degradation, and the positive charge of siRNA prevent its diffusion across cellular membranes during siRNA delivery (Kulkarni et al., 2019), which still needs to be addressed.

In this report, the aim was to construct a multifunctional GNR-based therapeutic system combined with pH-sensitive CPP to improve the uptake efficiency of cells and tumor targeting. Importantly, efficient delivery of siRNA (target lysosomes) significantly restrained the PTT-induced autophagy and then weakened the resistance of autophagy to PTT. Therefore, this study provides a potential platform for PTT to treat cancer with superior efficacy.

## Materials and Methods

### Chemical and Reagents

TFEB siRNA was designed by TSINGKE (Beijing, China). The sequences are as follows: 5’-: GAU​GUC​AUU​GAC​AAC​AUU​ATT and R: UAA​UGU​UGU​CAA​UGA​CAU​CTT-3’. Chloroauric acid (HAuCl4.4H2O), sodium chloride, hexadecyltrimethylammonium bromide (CTAB), l-Ascorbic acid, sodium borohydride (NaBH4), silver nitrate (AgNO3), l-ascorbic acid, poly (diallyldimethylammonium chloride) (PDDAC, MW 100,000–200,000 g/mol), and poly (sodium 4-styrenesulfonate) (PSS) were purchased from Aldrich (Shanghai, China). Hydrochloric acid (HCl) was obtained from Sinopharm Chemical Reagent Co., Ltd. (Shanghai, China). Cell-penetrating peptides [CPP, Pep-TH, AGYLLGHINLHHLAHL (Aib) HHIL-Cys] (Supplementary Figure S1A) were supplied by Shanghai Apeptide Co., Ltd. (Shanghai, China). More than 95% of the pure peptide was analyzed by LC-MS and 2467.90 of the molecule was analyzed by high-performance liquid chromatography (HPLC) (Supplementary Figures S1B,C).

### Synthesis of GNRs, GNR@siRNA, and GNR@siRNA/CPP

To synthesize the GNRs, a seed-mediated growth method (El-Sayed, 2003) was adopted. Briefly, under vigorous stirring, 0.6 ml of 10 mM fresh NaBH_4_ was immediately injected into 5 ml of 0.5 mM HAuCl4, and they were mixed with 5 ml of 0.2 M CTAB solution. The color changed from yellow to bright brown, and the stirring was stopped. This seed solution was aged at least 1 h before being used. A total of 15 ml (0.1 M) AgNO_3_ and 1.2 ml (5 mM) HAuCl4 were added to 6 ml (0.2 M) CTAB together. Afterward, 720 ml (10 M) Mascorbic acid and 15 ml (1.2 M) HCl were added to the solution and swirled until the color changed from dark orange to colorless. Then, 12 ml of the seed solution was added. Finally, the GNRs were harvested by centrifugation at 10,000 rpm for 10 min. In the synthesis of GNR@siRNA/CPP, the GNRs are scattered to 2 ml of 10 mg/mLPSS (dispersed in 1 mM NaCl) and stirred for 2 h at room temperature to get PSS-coated GNRs (GNR/PSS) by adopting a similar method to the PDDAC further coated. Subsequently, the GNRs/PSS/PDDAC were mixed with different amounts of siRNAs and incubated for 1 h to obtain GNR/PSS/PDDAC@siRNA (GNR@siRNA). In the synthesis of GNR@siRNA/CPP, the GNR@siRNA nanoplex was mixed with different amounts of CPP by pipetting and incubated for 24 h at room temperature.

### Cell Culture

Human OS cell lines (HOSs), U2-OS (U2), 143B, and MG-63 were purchased from the Cell Bank of the Chinese Academy of Sciences (Shanghai, China, cultured in Dulbecco’s modified Eagle’s medium) (DMEM), supplemented with 10% fetal bovine serum (FBS) and 1% antibiotics (penicillin–streptomycin). All cells were maintained at an appropriate atmosphere.

### Characterizations

The molecular weight and the purity of the sample were confirmed by Shimadzu LCMS-2020 and high-performance liquid chromatography (HPLC), respectively. Transmission electron microscopy (TEM, Hitachi H-7650) was used to observe the morphology of GNR nanoplatforms. The zeta potentials of the samples were investigated on a zetasizer (Malvern Instruments, UK), and the absorption spectra were performed on a TU-1810 UV-V spectrophotometer.

### Agarose Gel Electrophoresis

The GNR nanoplexes with different siRNA ratios were loaded onto 1% agarose gels with 0.5 mg/ml ethidium bromide. The gels were electrophoresed at 100 V (15 min) and acquired by using UV light on a Gel Doc 2000 imager system (Bio-Rad, Munich, Germany).

### *In Vitro* Photothermal Measurement

Cells were plated on 12-well tissue culture plates and incubated for 24 h, and the culture medium was then removed and replaced with a culture medium containing the conjugated nanoparticles. Afterward, each sample was exposed or unexposed to a near-infrared (NIR) laser (808 nm, Tengxing Electronic Technology Co., Ltd. Guangdong, China) at 1 W/cm^2^ for 5 min. Finally, a high-precision infrared thermal imager (ST9450, Xima, Guangzhou, China) was applied to monitor the temperature change.

### Cell Invasion and Migration Assays

Cells were seeded into the well plate with different treatment. After 24 h, the Cell Counting Kit-8 (Dojindo, Japan) dye was added to investigate cellular survival. For the wound-healing assay, a wound was made using a pipette tip, and when the cell proliferation reached approximately 80% confluence in the 6-well plate, it was used to detect cell migration. Transwell chambers (Corning Life Science, United States) were chosen to determine cell invasion and migration abilities.

### Measurement of Endocytosis

Cells were grown to about 80% confluence in 12-well plates and then incubated with siRNA (labeled with the green fluorescent FAM), GNR@siRNA, and GNR@siRNA/CPP for 24 h, respectively. After the culture medium was removed, a confocal laser microscope (Nikon, A1PLUS, Tokyo, Japan) was used to capture fluorescence images immediately, and the densities of the fluorescence intensity were analyzed using ImageJ software (NIH, Bethesda, MD, United States).

### Live/Dead Staining

Cells were seeded in well plates and grown to about 80%, treated with different nanoparticles for 24 h, and then irradiated under 808 nm NIR laser (1 W/cm2) for 5 min. Next, 2 μM calcein (Beyotime, China) and 50 μg/ml PI (Beyotime, China) were used to stain the cells for 10 min before observation by confocal laser microscopy (Nikon, A1PLUS, Tokyo, Japan).

### *In Vivo* Experiments

All animal experiments complied with the guidelines and followed a protocol that was approved by the Research Ethics Committee of Zhejiang University, China (Permit No. 2019/053). Nude mice (4-week-old, male) were obtained from the Shanghai Laboratory Animal Center (Chinese Academy of Science, Shanghai, China). The mice were raised under specific pathogen-free conditions and inoculated with 2 × 106 HOS cells *via* the marrow cavity of the right tibia and armpit subcutaneously. After 7 days, these mice were randomized into four groups. The mice received the GNR complex (10 mg/kg) and an equivalent volume of PBS (CON group) with a time interval of 4 days, respectively. Tibial axillary and armpit tumors were harvested 3 weeks after treatment, with each tumor being weighed. The tumor sizes were calculated as volume (cm^3^) = [π × width^2^ (cm^2^) × length (cm)]/6.

### Western Blotting

Cellular proteins (60 μg) were transferred onto a polyvinylidene fluoride membrane (Bio-Rad Laboratories), and after being blocked by 5% fat-free milk for 1.5 h, the membranes were incubated with the following primary antibodies: active caspase-3 (1:1,000, CST), LC3I/II (1:1,000, Proteintech Group, Inc.), and GAPDH (Cat: RT1210-1, Huabio) overnight at 4°C. Then, the membranes were incubated with a secondary antibody for 1 h. The signals were visualized with the ChemiDoc^TM^ XRS + Imaging System (Bio-Rad Laboratories, Hercules, CA, United States).

### Immunofluorescence

After the samples were fixed with 4% paraformaldehyde and blocked by 5% bovine serum albumin (BSA), the samples were incubated with the following primary antibodies: active caspase-3 (1:100, CST) and LC3B (1:200, Proteintech Group, Inc.) at 4°C overnight. Afterward, the samples were incubated with the secondary antibody (1:1,000, Huabio), and the sections were then restained with an anti-fluorescent quench sealant (Yeasen). The fluorescence images were captured by confocal laser microscopy (Nikon, A1PLUS, Tokyo, Japan), and the densities of the fluorescence intensity were analyzed using ImageJ software (NIH, Bethesda, MD, United States).

### Hematoxylin and Eosin

Tissues were harvested and fixed in 4% (w/v) paraformaldehyde for 24 h, after gradient alcohol dehydration and being embedded in paraffin wax. The sections were then prepared and mounted on slides for H&E and cresyl violet staining. Images were obtained by light microscopy.

### Statistical Analysis

All data were presented as means ± SEM. Differences between two groups were determined using Student’s t-test, whereas one-way ANOVA followed by Dunnett’s *post hoc* test was used to determine differences among multiple groups. *p* value < 0.05 was considered to be significant.

## Results and Discussion

### Synthesis and Characterization of the GNR@siRNA/CPP System

**FIGURE 1:**
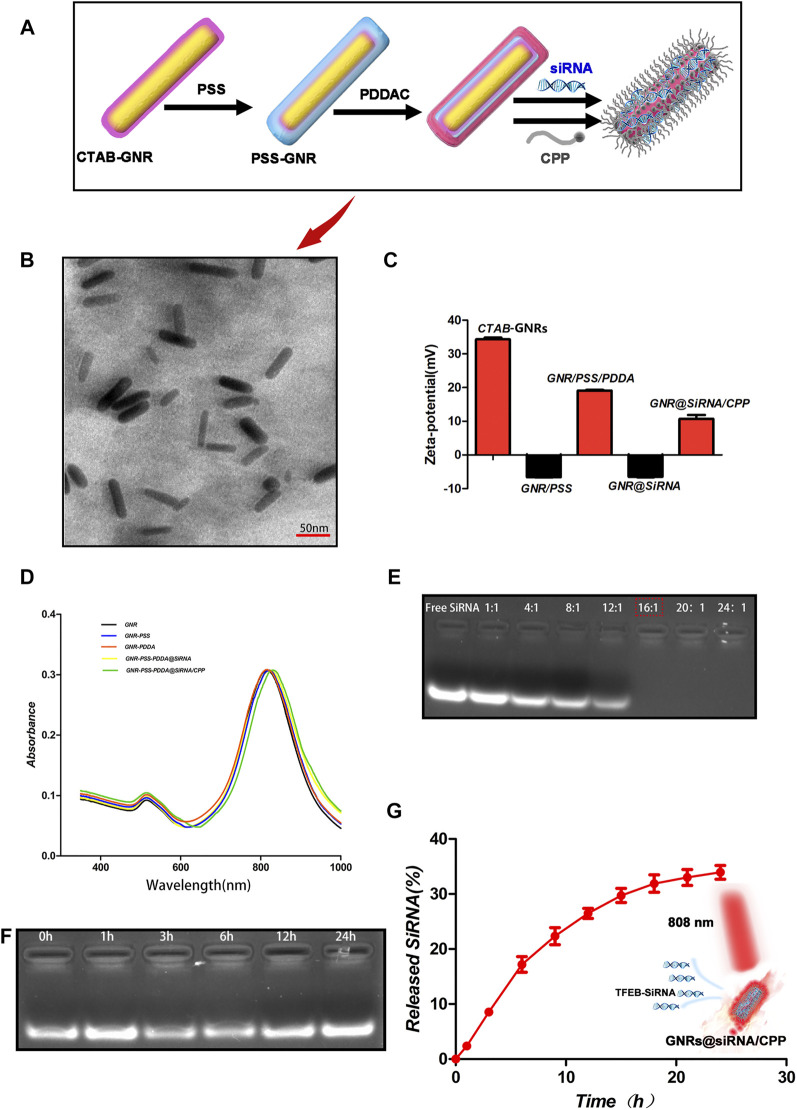
Characterization of the nanoplatforms. **(A)** Schematic illustration of the synthesis of GNR@siRNA/CPP nanorods. **(B)** TEM images of GNR@siRNA/CPP nanorods. **(C, D)** Zeta potential and UV-vis spectrum of GNRs, GNR-PSS, GNR-PSS-PDDAC, GNRs-PSS-PDDAC-siRNA (GNRs@siRNA), and GNR/PSS/PDDAC-siRNA-CPP (GNR@siRNA/CPP). **(E, F)** Agarose gel retardation assay of GNR/siRNA complexes under various weight ratios and protection of siRNA against fetal bovine serum digestion. **(G)** siRNA cumulative release from GNR@siRNA/CPP complexes at different points in time.

In this study, the GNR@siRNA/CPP delivery vehicle was acquired *via* a well-developed layer-by-layer assembly approach. Before PDDA (positively charged) coating, the original CTAB on the surface of GNRs was replaced by the sodium polystyrene sulfonate (query) (PSS, negatively charged) layer. Meanwhile, the PDDA made the siRNA electrostatic bond with GNRs. Next, CPP was immobilized on the GNR surface by sulfur–gold bonds (Figure 1A). As revealed by TEM (Figure 1B), the GNR@siRNA/CPP indicated a regular rod-shaped and well-dispersed character, and being proved after modification and conjugation, GNRs were still in a well-dispersed status. The zeta potential values of the CTAB–GNRs, PSS-GNRs, PSS-PDDA–GNRs, PSS-PDDA–GNR@siRNA, and PSS-PDDA–GNR@siRNA/CPP were + 34.3, −6.5, + 19.1, −6.4, and + 10.7 mV, respectively (Figure 1C). After the incubation of the cationic GNR/PSS/PDDAC substrate with anionic siRNA, the zeta potentials reverse from positive to negative, while that after the anionic GNR/PSS/PDDAC/siRNA substrate with cationic CPP, the zeta potentials reverse from negative to positive, which suggests the binding of CPP with GNRs-siRNA in the final GNRs-siRNA/CPP. Being attributed to the siRNAl and CPP doping, the GNR complex exhibited a continuous red shift that was observed from the longitudinal SPR (LSPR) in the absorption spectra (Figure 1D). Based on the above experimental results, we concluded that the siRNA and CPP were successfully bound to the surface of GNRs.

**FIGURE 2:**
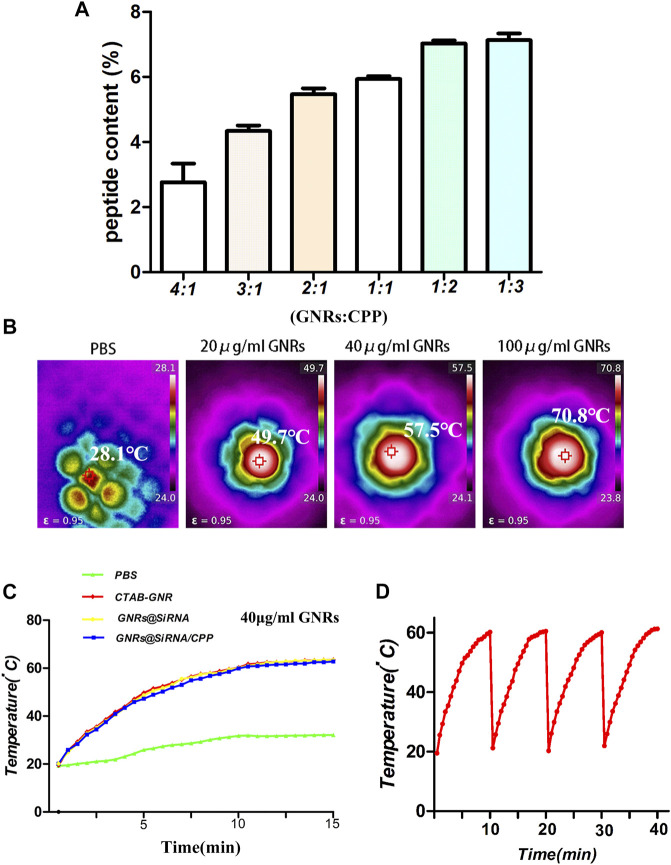
Characterization of the GNR/CPP complexes. **(A)** Peptide content on GNR/CPP complexes surface under various weight ratios. **(B)** Temperature plot of different concentrations of GNRs irradiated by 808 nm laser irradiation (1 W/cm^2^). **(C)** Temperature changes of GNRs and GNR complexes irradiated by 808 nm NIR laser. **(D)** Temperature change curves of the GNR@siRNA and GNR@siRNA/CPP irradiated by 808 nm laser.

Two important factors, the binding stability and release of siRNA, needed to be considered before the siRNA delivery platform was performed. The agarose gel electrophoresis assay was used to investigate the optimal binding ratio of GNRs to TFEB siRNA. As shown in Figure 1E, when the mass ratio of GNRs to siRNA is 16:1, siRNA almost completely binds to GNRs. And then, the GNR@siRNA was incubated in 50% fetal bovine serum (FBS) at 37°C for different time periods, and after 24 h of incubation, the particle-bound siRNA still remained intact, which confirms the stability of the delivery system siRNA that binds to GNRs. The released siRNA was also tested from the GNR@siRNA/CPP *via* the absorbance of the supernatant at 260 nm. After 24 h, the accumulated siRNA release reached about 35% (Figure 1G) that was enough to make the biological availability. The Micro BCA assay was used to measure the peptide contents on the GNRs (Figure 2A). When the ratio between the peptide and GNRs was greater than 1:1, about 6% of the total weight of GNR@siRNA/CPP can provide excellent biological functions. In addition, the photothermal conversion ability of the GNR complex was also tested. Various concentrations of GNR complexes can be heated from room temperature to about 49.7–70.8°C after 5 min irradiation under an 808 nm laser (Figures 2B,C), and all GNR complexes can realize ternary heating–cooling cycle for the excellent photothermal stability (Figure 2F).

### Cellular Uptake of the GNR@siRNA/CPP System.

**FIGURE 3:**
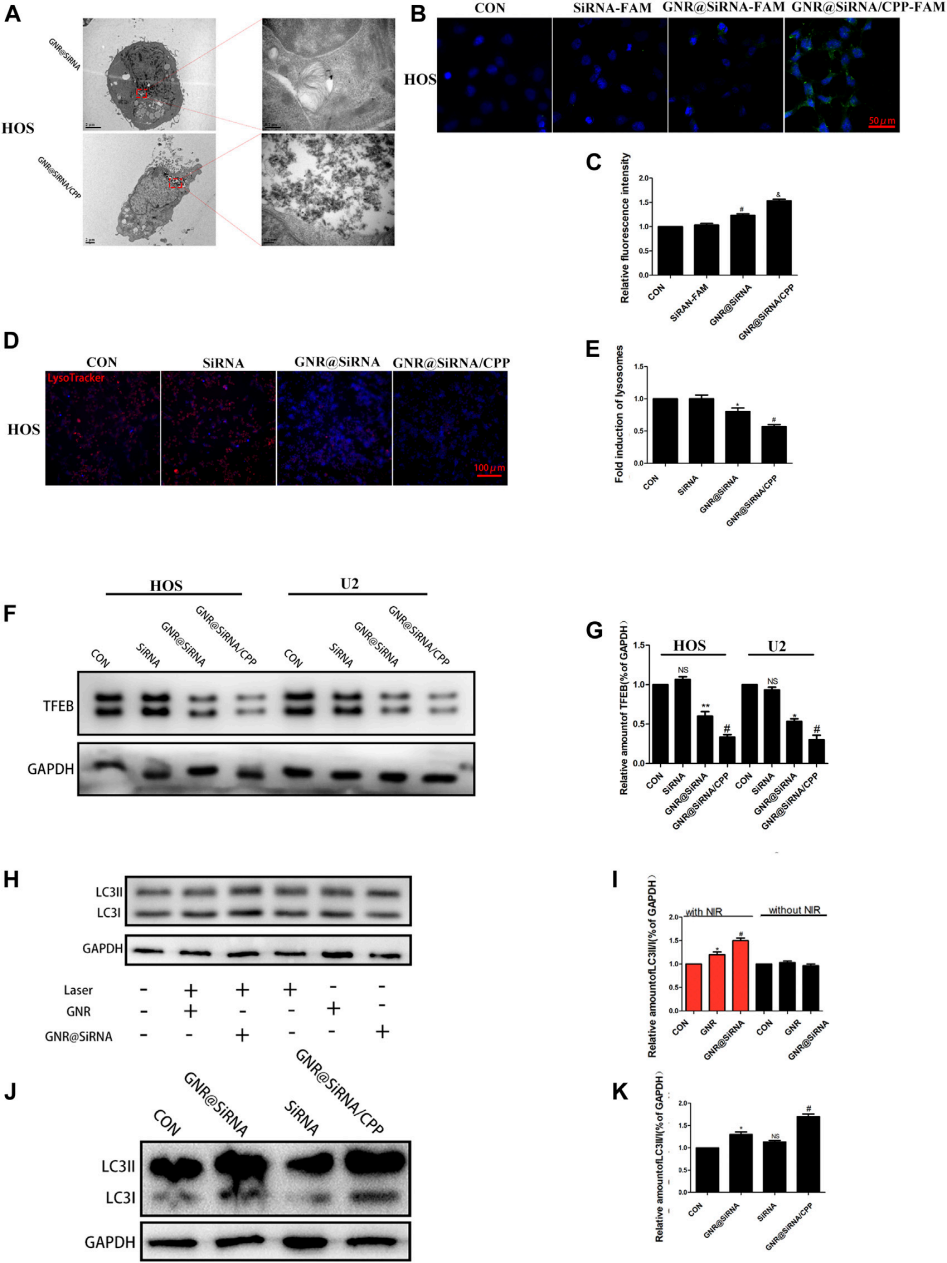
Nanoplatforms inhibits lysosome formation. **(A)** TEM images of GNR@siRNA and GNR@siRNA/CPP in OS cells. **(B, C)** Fluorescent images of OS cells incubated with siRNA (labeled with the green fluorescent FAM), GNR@siRNA, and GNR@siRNA/CPP, respectively. **(D, E)** Confocal microscopic images of Lyso Tracker for lysosomes (red) in OS cells. **(F, G)** Protein expression and densitometric quantification of TFEB in different groups. **(H–K)** Protein expression and densitometric quantification of LC3B in different groups. (*n* = 5 per group; *p < 0.05 vs. CON group, **p < 0.01 vs. siRNA group, ^**#**^ *p* < 0.05 vs. siRNA group or GNR@siRNA group; ^&^ *p* < 0.05 vs. GNR@siRNA group).

 The pharmacokinetic and biodistribution of nanoplatforms need to be considered before treatment. It has been reported that coated gold nanorods were injected intravenously in mice, and their concentration peaked in the blood about 1 h later. After 24 h, they were basically cleared and gradually increased in tissues and organs. Meanwhile, they could still be detected after 24 h, mainly in the spleen, liver, and tumor tissues (Arunkumar et al., 2015; Puleio et al., 2020). The cellular uptake of GNR@siRNA/CPP was detected in HOS and U2 osteosarcoma cell lines. As illustrated in Figures 3B,C and Supplementary Figures S2A,B, FAM siRNA was observed in GNR complex groups by laser confocal, and the fluorescence intensity follows the hierarchy: GNR@siRNA/CPP > GNR@siRNA > GNRs > CON group. TEM images were used to visualize the nanorods in the cells, and the results (Figure 3A; Supplementary Figure S2C) showed the same results as laser confocal. These results suggested that CPP increased the amount of GNR complex cellular uptake by OS cells. To further explore the effectiveness of siRNA vectors, the expression of the target protein TFEB and the lysosomes was detected in OS cells. As anticipated, free siRNA did not have effects on the expression of TFEB protein levels; the GNR complexes inhibited the expression of TFEB protein, and the GNR complex with CPP showed stronger inhibition in OS cells (Figures 3F,G). The LysoTracker red staining assay was conducted to investigate the number of lysosomes in OS cells. As demonstrated in Figures 3D,E and Supplementary Figures S2D,E, the number of lysosomes in OS was unaffected by free siRNA, and the GNR complexes inhibited the lysosomal formation in OS cells. The autophagy-related protein LC3B was also investigated in OS cells. As displayed in Figure 3K, the expression of LC3B was significantly increased in the GNR group, which was reversed by the GNR@siRNA group under NIR irradiation. However, it did not have that trend without NIR irradiation in OS cells. Additionally, the free siRNA did not inhibit the LC3B protein expression, and compared with the GNR@siRNA group, the GNR@siRNA/CPP group exerted much effective ability for the promotion of the expression of LC3B protein in OS cells (Figures 3H–K; Supplementary Figures S2F–I). Therefore, it was concluded that an siRNA delivery vector that can efficiently transport siRNA and realize the efficient gene silencing of the target gene was constructed successfully.

### *In Vitro* Photothermal Effects

**FIGURE 4:**
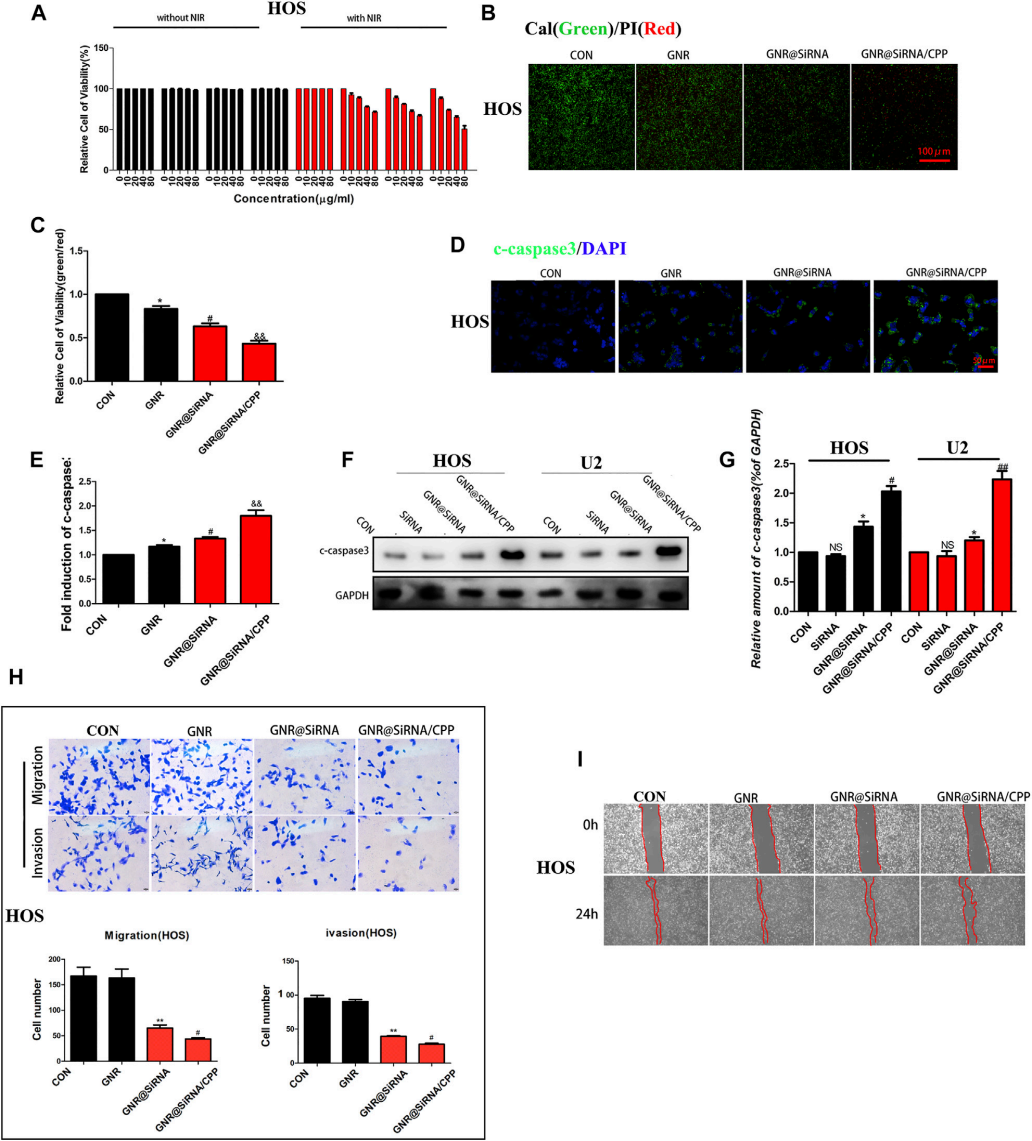
Nanoplatforms inhibit OS cell activity *in vitro*. **(A)** Cell-survival rate in OS cells. **(B, C)** Living/dead staining of OS cells incubated with 100 μg/ml GNRs and GNR complexes for 24 h and then irradiated under 808 nm NIR laser (1 W/cm^2^). **(D, E)** Immunofluorescence staining of c-caspase-3 in different groups. **(F, G)** Western blotting and densitometric quantification. **(H)** Migration and invasion assay in different groups. **(I)** Wound-healing assay in different groups. (*n* = 5 per group; **p < 0.01 or ***p < 0.001 vs. CON group; ^**##**^ *p* < 0.01 vs. GNR@siRNA group; ^#^ *p* < 0.05 vs. GNR@siRNA group).

The cytotoxicities of GNR complexes on OS cells were first investigated by the cell survival rate *via* the CCK8 assay. As shown in Figure 4A; Supplementary Figures S3A–C, GNR complexes have no effects on the survival rate of OS cells without laser irradiation, suggesting that the GNR complexes are non-toxic themselves. When OS cells were exposed to GNR complexes, the survival decreased was dose-dependent under laser irradiation conditions. Furthermore, due to higher cellular uptake of GNR@siRNA/CPP, the GNR@siRNA/CPP group had better ability to kill tumor cells, which was further confirmed by Cal/PI double staining and Western blot for the apoptotic protein (Figures 4B,C; Supplementary Figures S3D,G). Hence, it can be concluded that gene silencing by GNR complexes and photothermal effects can synergically kill OS cells, and CPP increases GNR complex entry into tumor cells and promotes apoptosis. Most cancer-related deaths are attributable to metastases, but the current clinical treatments are unsatisfactory (Liao et al., 2021). Transcription factor EB (TFEB) links autophagy to lysosomal biogenesis (Settembre et al., 2011) and promotes metastasis in cancer (Liang et al., 2018; He et al., 2019). Moreover, autophagy has been confirmed to be involved in modulating tumor cell motility and invasion (Sharifi et al., 2016). Hence, the experiment was performed to determine whether GNR complexes have effects on the migration of OS cells, and the GNR complexes were exposed to the OS cells under 808-nm near-infrared (NIR) light, where the temperature was regulated without affecting the cell viability/proliferation. Our results demonstrated that the invasion and migration of the GNR complex groups were significantly abrogated in OS cells, compared with those in CON and GNR groups (Figure 4H; Supplementary Figures S3H–J), which was similar to the results observed in the wound-healing assay (Figure 4I; Supplementary Figure S3K), suggesting that the nanocarrier successfully transported the interfering gene with inhibitory effects on OS cells *in vitro*.

### *In Vivo* Experiment

**FIGURE 5:**
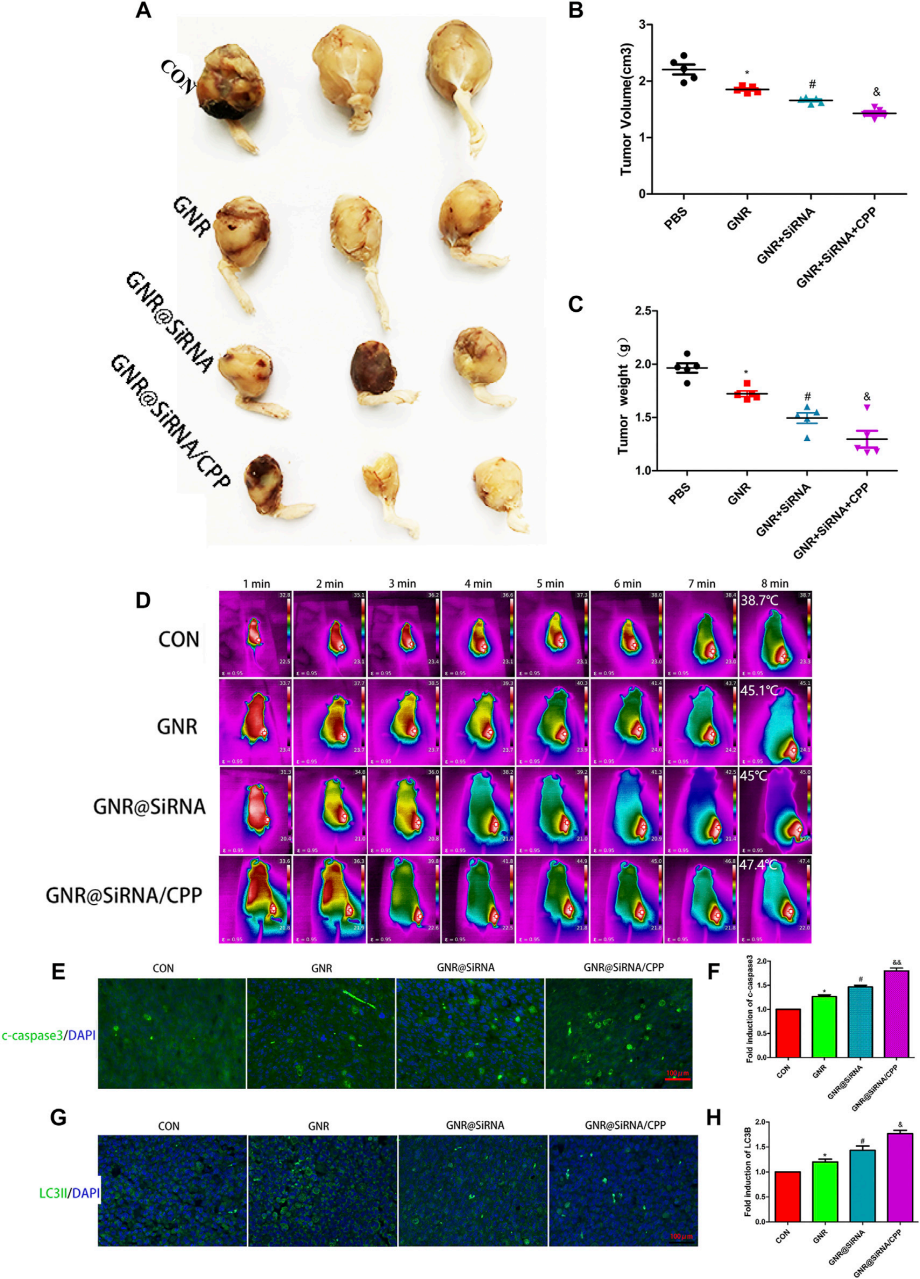
Nanoplatforms inhibit the growth of the marrow cavity of OS *in vivo*. **(A–C)** Photographs of harvested xenograft tumors in different groups. **(D)** Thermographs of mice recorded in different groups by NIR irradiation for various times. **(E–H)** Immunofluorescence staining of c-caspase3 and LC3B in different groups. (*n* = 5 per group; *p < 0.05 vs. CON group; ^**#**^ *p* < 0.05 vs. GNR group; ^&^ *p* < 0.05 or ^&&^ *p* < 0.01 vs. GNR@siRNA group).

**FIGURE 6:**
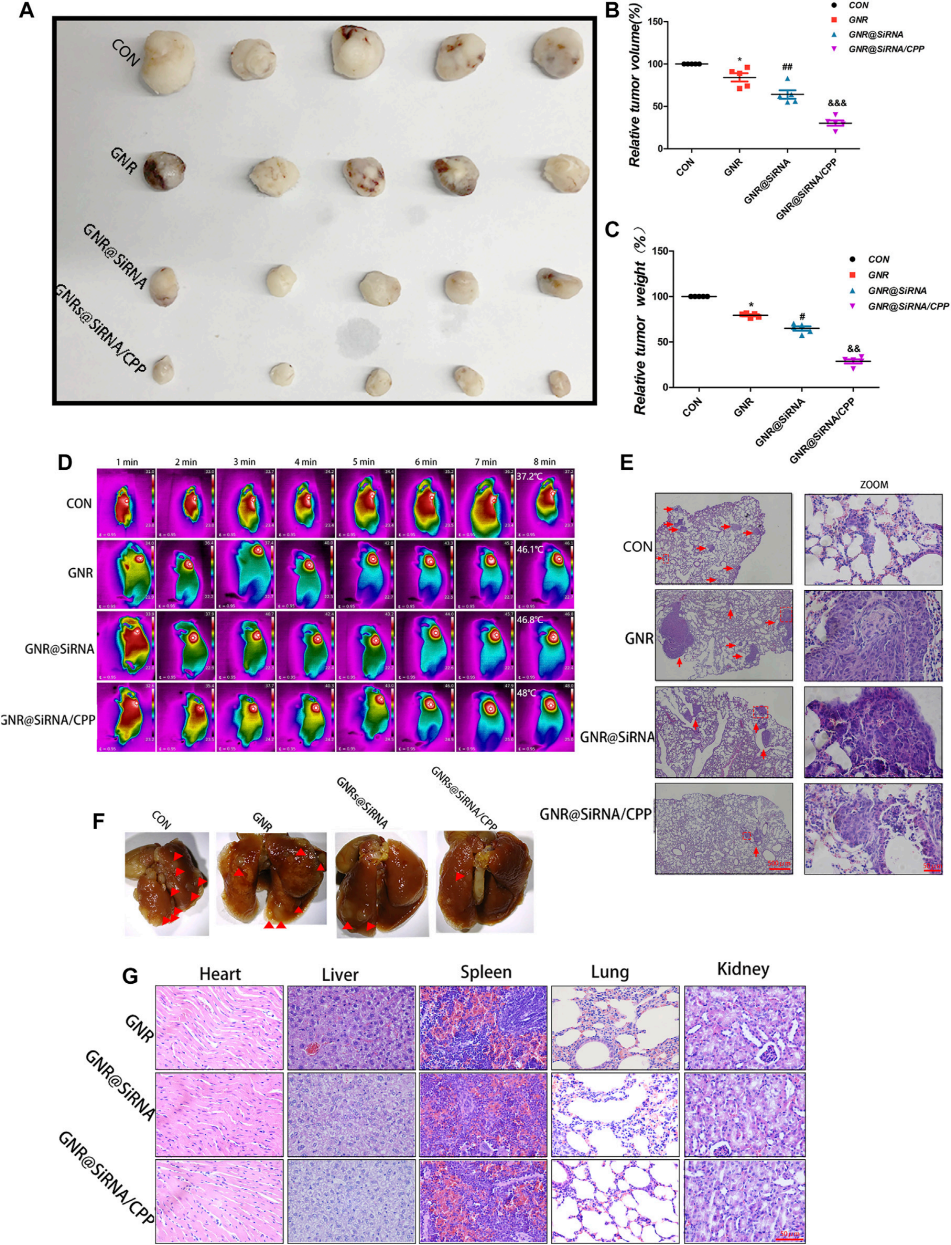
Nanoplatforms inhibit the growth of OS subcutaneously and pulmonary metastasis *in vivo*. **(A–C)** Tumor formation in different groups. **(D)** Thermographs of mice recorded in different groups by NIR irradiation for various times. **(E, F)** Photographs and H&E staining of lung tissues in different groups. **(G)** H&E staining of organ tissues of mice in different groups. (*n* = 5 per group; *p < 0.05 vs. CON group; ^**#**^ *p* < 0.05 or ^**##**^ *p* < 0.01 vs. GNR group; ^&&^ *p* < 0.01 or ^&&&^ *p* < 0.001 vs. GNR@siRNA group).

To further verify the efficacy of the GNR complex for OS, we tested *in situ* and subcutaneous mouse models of OS. In *in situ* mouse models, the GNR complex decreases the growth of the tumors compared with that of the control group and decreases the growth of the tumors with the following hierarchy: GNR@siRNA/CPP > GNR@siRNA GNRs > CONgroup (Figures 5A–C). Being encouraged by the antitumor efficacy of the GNR complex *in vivo*, it was necessary to evaluate the conversion property of nanocomposites under NIR light *in vivo*. We investigated the photothermal therapy of GNR complexes by performing with an 808 nm laser. After irradiation for 8 min, the tumor area in CON, GNR, GNR@siRNA, and GNR@siRNA/CPP showed temperatures of 38.7°C, 45.1°C, 45°C, and 47.4°C, respectively (Figure 5D), which were enough to achieve the effects of tumor ablation in the GNR and GNR complex groups. pH-sensitive CPP increased the GNR complex into the tumor cells, thus leading to sufficient tumor ablation. In subcutaneous mouse models, the GNR complex presented the same inhibiting tumor growth tendency and photothermal conversion ability with that *in situ* mouse models (Figures 6A–D). Next, the cleaved caspase-3 and LC3B-positive cells in the tumor tissue were detected. The GNR@siRNA/CPP group resulted in a more remarkable increased apoptosis of tumor cells than other groups (Figures 5E,F). The hierarchy of the amount of LC3B-positive cells is as follows: GNR@siRNA/CPP > GNR @siRNA > GNRs > CON group (Figures 5G,H). This indicated that the silencing of the TFEB gene by the GNR complex reduced the generation of lysosomes and the binding between lysosomes and autophagosomes, thereby leading to the blocked autophagy. LC3B accumulation increases the photothermal effects and tumor cell apoptosis.

*In vitro*, we have demonstrated that GNR complexes abrogated the invasion and migration of OS cells, and some studies have revealed that patients with osteosarcoma had evidence of metastases at diagnosis, mostly in the lungs (Meazza and Scanagatta, 2016). Therefore, we examined the influences of the GNR complex on lung metastasis of OS *in vivo*. As presented in Figures 6E,F, the lungs of the CON group exhibited multiple small metastatic tumor nodules which increased compared with the GNR complex group, and the hierarchy of the GNR complex inhibited lung metastasis of OS as follows: GNR@siRNA/CPP > GNR @siRNA > GNRs > CON, indicating that the nanocarrier is successful in transporting cargoes and facilitating biological functions of the cargo *in vivo*. The biosecurity of the GNR complex was also detected by the H&E-stained vital organs, and no obvious damage was observed in H&E-stained histopathological slices of the heart, liver, spleen, lungs, and kidneys (Figure 6G). In summary, the GNR complex was designed and synthesized by exerting an admirable ability to carry siRNA, and pH-sensitive CPP increased the tumor-targeting ability of the GNR complex, which plays a synergistic role in gene silencing and photothermal ablation of tumor *in vivo* experiments.

## Conclusion

In this study, the GNR@siRNA/CPP nanoplatforms were designed to efficiently deliver siRNA oligos targeting TFEB so as to promote photothermal therapy. The multifunctional nanoplatforms exhibited excellent biocompatibility, photothermal conversion property, and stability. The GNR conjugation with pH-sensitive CPP resulted in the uptake efficiency of the nanoplatforms by OS cells, and tumor targeting was improved. In addition, the multifunctional nanoplatforms provide an effective protection of siRNA from unexpected degradation and a stable release rate. It was demonstrated that TFEB-siRNA delivered efficient intracellularly by GNR significantly, weakened the formation of autolysosomes, induced the inhibition of autophagy in OS cells, and then weakened the resistance of autophagy to PTT. Most importantly, the GNR@siRNA/CPP nanoplatforms exerted efficacy to suppress metastasis of OS. Both *in vitro* and *in vivo* anti-osteosarcoma effects were remarkably enhanced, which attributes to the synergistic effects by GNR@siRNA/CPP nanoplatforms under NIR irradiation conditions. Therefore, this study indicated a potential multifunctional nanocarrier system for tumor therapy and metastasis.

## Data Availability

The original contributions presented in the study are included in the article/Supplementary Material; further inquiries can be directed to the corresponding authors.

## Supplementary Material

Supplementary Figure S1 Characterization of the GNR/CPP complexes. **(A)** Structural diagram of the cell-penetrating membrane peptide (CPP). **(B, C)** Molecular weight of CPP detected by MS and the purity of CPP detected by HPLC.

Supplementary Figure S2 Nanoplatforms inhibit lysosome formation. **(A, B)** Fluorescent images of OS cells incubated with siRNA (labeled with the green fluorescent FAM), GNR@siRNA, and GNR@siRNA/CPP, respectively. **(C)** TEM images of GNR@siRNA and GNR@siRNA/CPP in OS cells. **(D, E)** Confocal microscope images of Lyso Tracker for lysosomes (red) in OS cells. **(F–I)** Protein expression and densitometric quantification of LC3 in different groups. (*n* = 5 per group; **p* < 0.05 vs. CON group, ***p* < 0.01 vs. siRNA group, ^**#**^ *p* < 0.05 vs. siRNA group or GNR@siRNA group; ^**&**^ *p* < 0.05 vs. GNR@siRNA group).

Supplementary Figure S3 Nanoplatforms inhibit OS cell activity *in vitro*. **(A–C)** Cell survival rate in OS cells. **(D, G)** Living/dead staining of OS cells incubated with 100 μg/ml GNRs and GNR complexes for 24 h and then irradiated under 808 nm NIR laser (1 W/cm^2^). **(E, F)** Immunofluorescence staining of c-caspase3 in different groups. **(H–J)** Migration and invasion assay in different groups. **(K)** Wound-healing assay in different groups. (*n* = 5 per group; ***p* < 0.01 or ****p* < 0.001 vs. CON group; ^**##**^ *p* < 0.01 vs. GNR@siRNA group; ^**#**^ *p* < 0.05 vs. GNR@siRNA group).
