# Supplementary figures and images for: Delivery of siRNA Using Functionalized Gold Nanorods Enhances Anti-Osteosarcoma Efficacy
Source: Front Pharmacol. 2021 Dec 20;12:799588. doi: 10.3389/fphar.2021.799588 (PMC8721171; doi:10.3389/fphar.2021.799588)

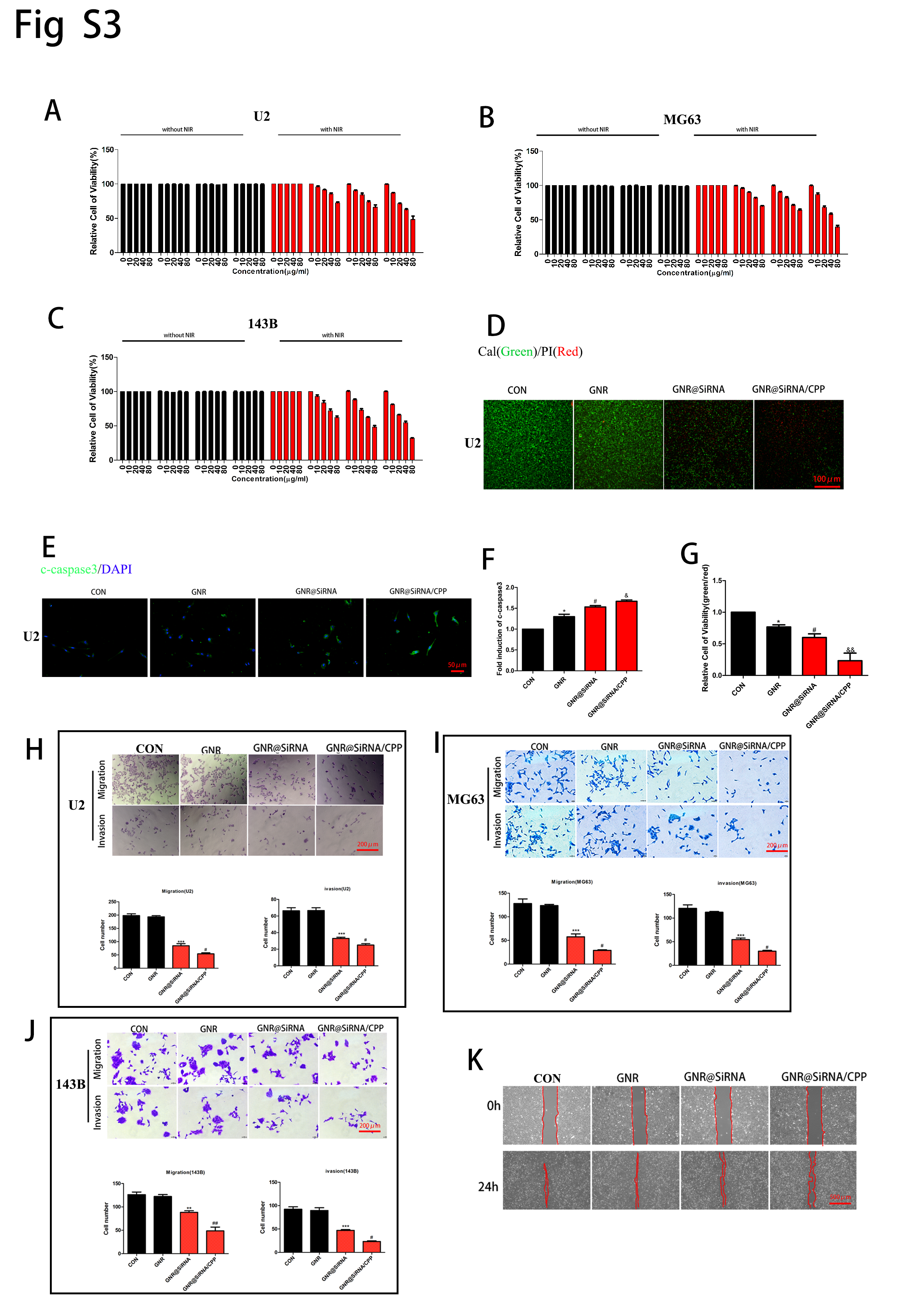

Supplement: Supplementary file 1 [file Image3.tif]

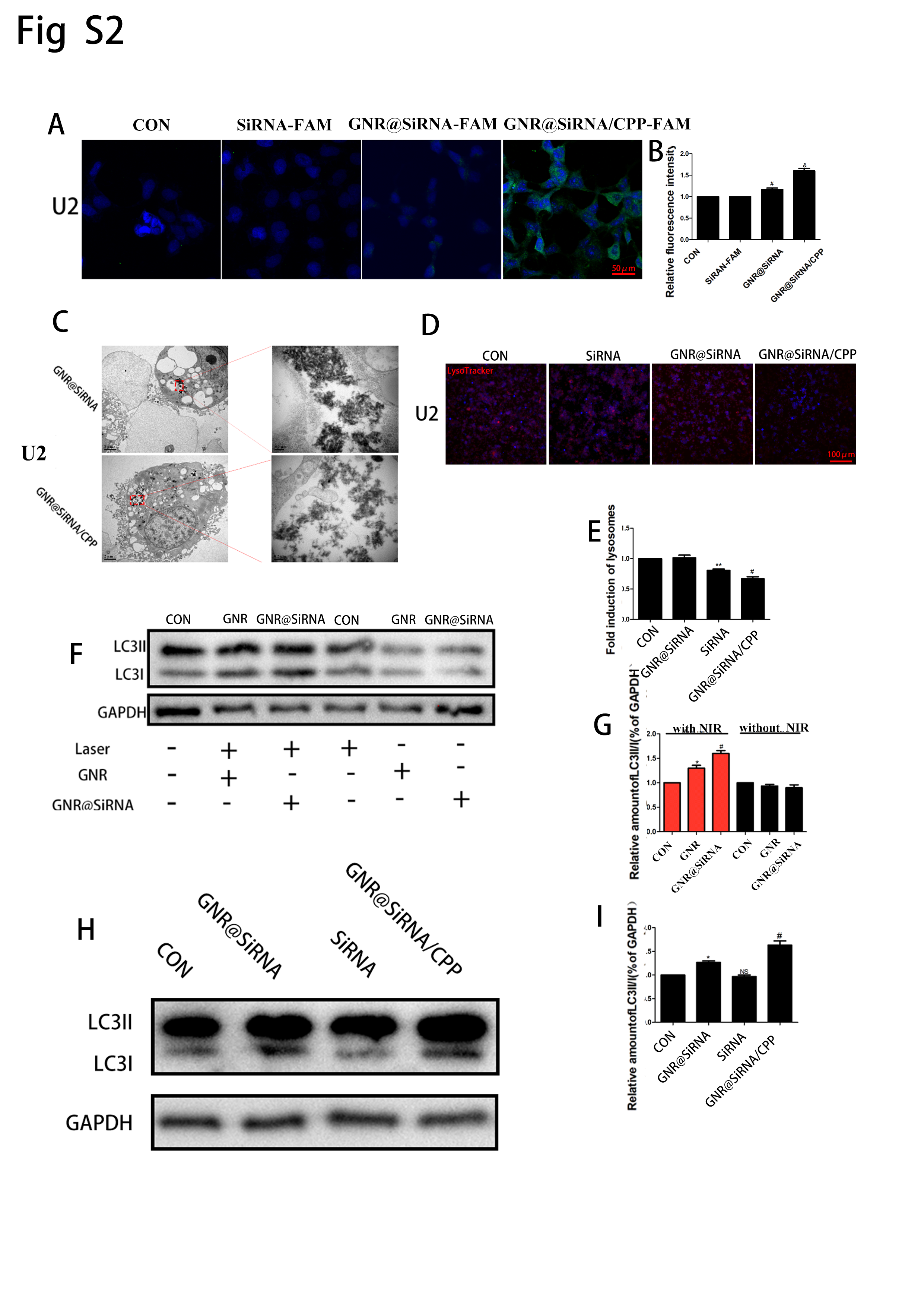

Supplement: Supplementary file 2 [file Image2.tif]

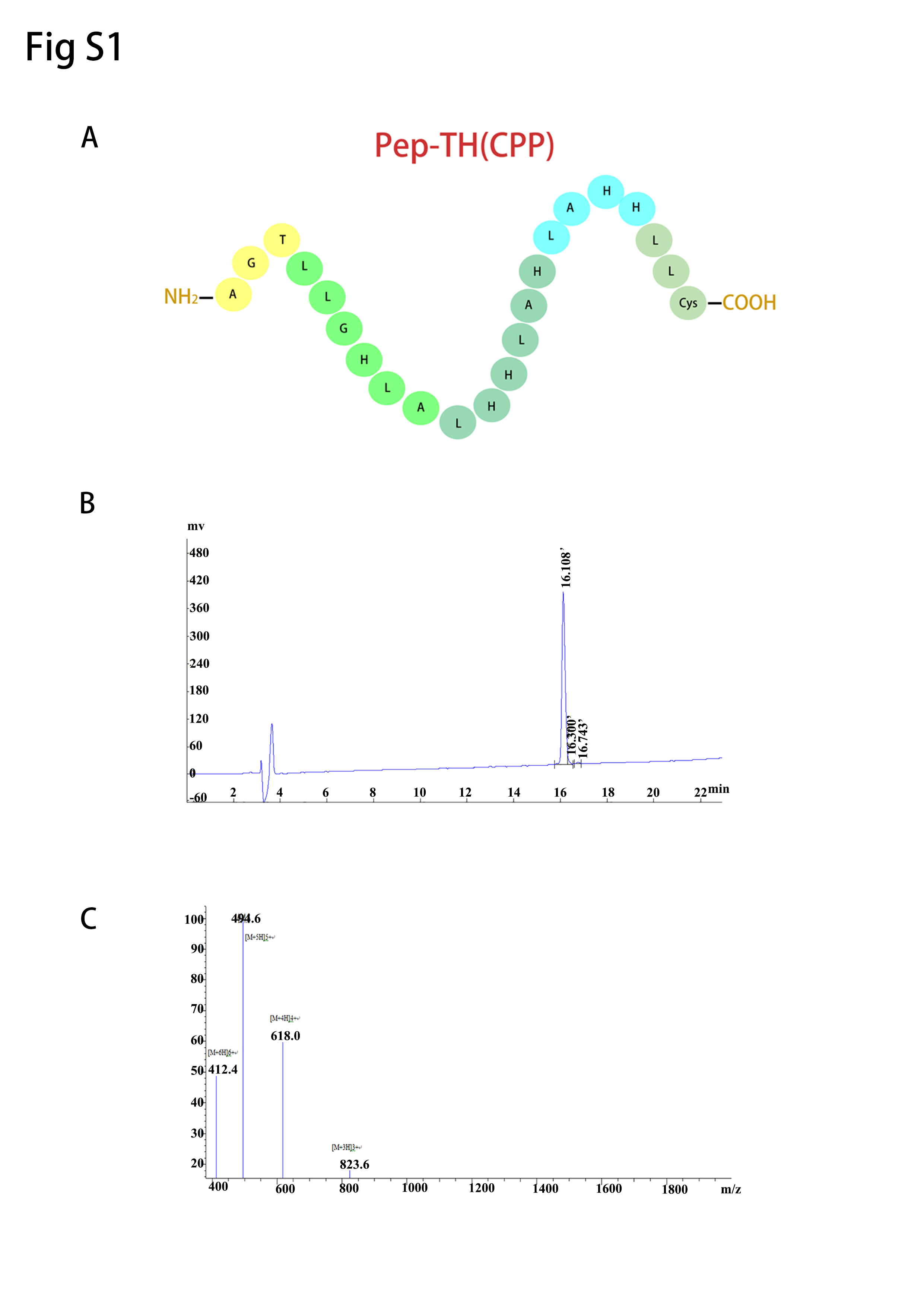

Supplement: Supplementary file 3 [file Image1.tif]
